# Supplementary figures and images for: Prenylquinones in Human Parasitic Protozoa: Biosynthesis, Physiological Functions, and Potential as Chemotherapeutic Targets
Source: Molecules. 2019 Oct 16;24(20):3721. doi: 10.3390/molecules24203721 (PMC6832408; doi:10.3390/molecules24203721)

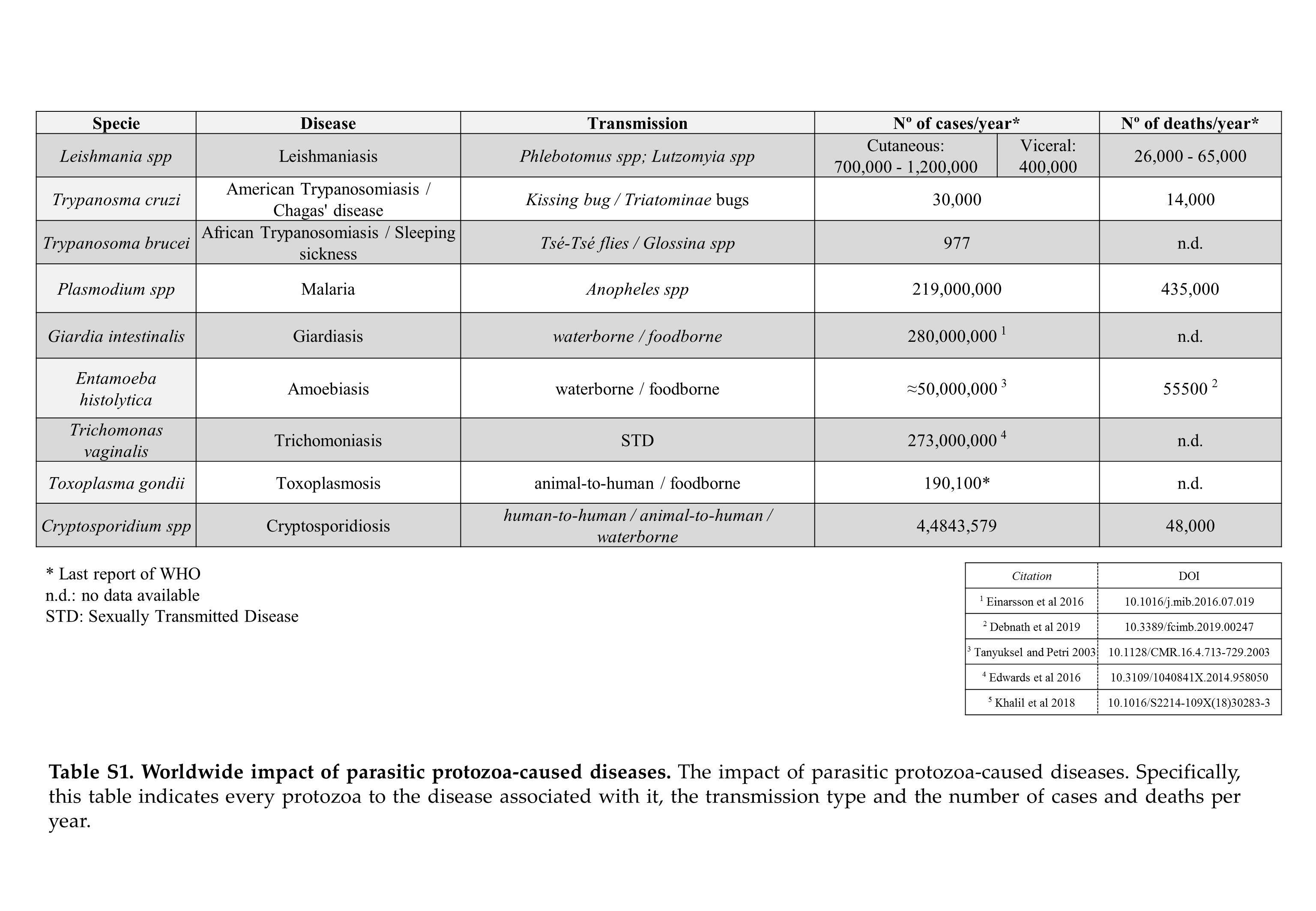

Supplement: Supplementary file 1 [file molecules-24-03721-s001.tif]
